# Transvaginal Ultrasound-Guided Core Biopsy—Experiences in a Comprehensive Cancer Centre

**DOI:** 10.3390/cancers13112590

**Published:** 2021-05-25

**Authors:** Dániel Lengyel, Ildikó Vereczkey, Krisztina Kőhalmy, Kiarash Bahrehmand, Zoltán Novák

**Affiliations:** 1 Department of Gynaecology, National Institute of Oncology, 1122 Budapest, Hungary; bahrehmand.kiarash@oncol.hu (K.B.); novak.zoltan@oncol.hu (Z.N.); 2 Doctoral School of Clinical Medicine, University of Szeged, 6720 Szeged, Hungary; 3 Department of Surgical and Molecular Pathology, National Institute of Oncology, 1122 Budapest, Hungary; vereczkey.ildiko@oncol.hu; 4 Department of Biochemistry, National Institute of Oncology, 1122 Budapest, Hungary; kohalmyk@oncol.hu

**Keywords:** transvaginal, sonography, tru-cut biopsy, core biopsy, ultrasound-guided, gynaecological cancer, diagnosis, TVUS

## Abstract

**Simple Summary:**

Adequate histological diagnosis defines the treatment in gynaecologic oncology. Although transvaginal ultrasound (TVUS) has widespread use in the diagnosis of pelvic tumours, TVUS-guided core biopsy is not a common procedure. In this study we summarize our experience in TVUS-guided biopsies performed in a comprehensive cancer centre, discussing the results of 303 patients who underwent this procedure. In addition, we compare the histological results of the biopsies with subsequent surgical histological results in 94 cases. Our study demonstrates that TVUS-guided core biopsy is a safe and effective histological sampling procedure providing adequate tissue for pathological evaluation in 99% of cases. Compared to surgically obtained histology, this procedure can reliably guide therapy, as its performance is satisfactory. In our opinion, TVUS-guided core biopsy is an effective diagnostic method providing possible benefits to patients referred for suspicion of gynaecological malignancy.

**Abstract:**

In this paper, we report our experience of transvaginal ultrasound (TVUS)-guided core biopsies involving 303 patients referred to the gynaecological ultrasound unit of our national comprehensive cancer centre. Adequate histologic specimens were obtained in 299 patients (98.7%). The most common sites of biopsy sampling were the adnexa (29.7%), the vaginal stump or wall (13.5%), the uterus (11.6%) and the peritoneum (10.2%). Malignancy was confirmed in two-thirds of patients (201/303) and a primary malignancy was diagnosed in 111 of the 201 histologically verified malignant cases (55.2%). Interestingly, 23.9% (48/201) of malignant tumours were proven to have a non-gynaecological origin. Among them, gastrointestinal tumours occurred the most frequently (31/48 patients). Three abscesses were discovered following the biopsy procedure, resulting in a complication rate of 1%. In 94 (31%) patients, subsequent surgery allowed the comparison of the ultrasound-guided and surgically obtained histologic results. We found inaccuracy in 12 cases (12.8%), which is discussed in this paper in detail. Sensitivity, specificity, PPV and NPV to diagnose malignancy was 94.8%, 94.1%, 98.7% and 80.0%, respectively. This is the largest study reported to date about the efficacy and safety of TVUS-guided core biopsy in evaluating pelvic lesions giving rise to a suspicion of gynaecological cancer.

## 1. Introduction

Histological diagnosis of female pelvic tumours is essential in their adequate and early clinical management. Ultrasound-guided biopsy is a routine diagnostic method to obtain tissue samples and used widely in different types of tumours [1,2,3]. Pelvic solid masses can be biopsied via transabdominal, transvaginal, transrectal or transperineal routes [4,5,6,7,8,9,10]. Generally, lesions located deep within the female pelvis are not easy to access transdabdominally due to various bowel loops, major vessels, uterus, urinary bladder and ureter being in the path of the needle [1,11,12]. In contrast, the transvaginal and transrectal approaches may provide a safer pathway for biopsy sampling, avoiding injury of the critical organs [12,13,14,15]. Adnexal masses may also be safely biopsied by this technique, offering a less-invasive diagnostic alternative to surgically obtained histology [5,16,17]. Transvaginal ultrasound (TVUS)-guided core needle biopsy (CNB) has several advantages: it can be an outpatient procedure, can be performed after a simple ultrasound examination and requires no preoperative preparation or fasting, enabling quicker histological diagnosis and possibly faster referral to definitive treatment [5,18,19]. Moreover, a recent study has demonstrated in female pelvic tumours that a more adequate biopsy rate was obtained through the transvaginal route compared to the transabdominal one [19]. Few papers analysed the accuracy and safety of TVUS-guided CNB in gynaecological cancer patients [14,18,19,20]. These authors demonstrated that it is a safe and effective diagnostic tool making an adequate histological diagnosis possible. Compared to fine-needle aspiration (FNA), the determination of a histological diagnosis may be facilitated due to the integrity and size of the specimen obtained by CNB [18,21]. For most certain solid tumours, CNB has higher sensitivity, specificity and diagnostic accuracy than FNA [21]. Diagnostic laparoscopy or explorative laparotomy is commonly performed to obtain definitive histological diagnoses in gynaecological cancer patients. In recurrent gynaecological cancer patients or primary advanced-stage ovarian cancer patients, surgery is no longer advocated if optimal cytoreduction is not feasible [1,22]. In general, ultrasound-guided CNB provides a less invasive way to obtain tissue samples for diagnosis, avoiding much greater invasiveness of laparoscopic or open surgical biopsy [14,16,23,24]. Despite its simplicity, this method is infrequently used in gynaecological oncology with few centres performing it regularly.

In this paper, we present our experience of TVUS-guided CNB of 303 patients referred to the gynaecological ultrasound unit of our national comprehensive cancer centre. Furthermore, we overview the indication and analyse the adequacy of the procedure.

## 2. Materials and Methods

All examinations of the prospective study were performed in our Department of Gynaecology at the Hungarian National Institute of Oncology. All patients who underwent a transvaginal, ultrasound-guided CNB sampling between March 2019 and December 2020 were included in the study. The majority of patients were referred to a histological verification with suspicion of gynaecological malignancy. A minority of the patients had biopsies for ruling out malignancy. Usually, patients with a suspicion of early-stage, non-disseminated ovarian cancer were not biopsied, as spillage is almost inevitable in cases of ovarian needle core biopsies. Only those patients are biopsied where intraabdominal dissemination is already suspected by imaging to avoid potential upstaging from organ-confined to non-organ-confined disease. However, there were 3 patients where uni- or multilocular cystic lesions were biopsied to avoid surgical exploration; in these cases, our main priority was to reduce invasivity due to advanced age and poor general health. Finally, in all cases, histology confirmed a benign disease. The predefined experimental parameters were continuously recorded and a prospective database was created.

All patients underwent a transvaginal ultrasound examination (Initially Aloka^®^, Tokyo, Japan, ProSound Alpha 6 ultrasound equipment with an Aloka^®^ UST-9124 180 Degrees 90R Probe ultrasound transducer, from September 2020 a Samsung^®^ Seoul, Korea, Hera W9 diagnostic ultrasound system with an EV3-10B transducer). When a lesion requiring histological confirmation was found, a core biopsy sampling was performed with a biopsy gun (BARD^®^ MAGNUM^®^ Reusable Core Biopsy System, Atlanta, GA, USA) using an Aloka UST-9124 or a Samsung JEM-063 stainless steel biopsy needle guide that could be applied to the transducer with an 18G × 30 cm needle. The penetration depth was set to 22 mm. A 15 mm penetration depth was used in some selected cases with an increased risk of a large vessel or ureteral injury. In all except 19 cases, we aimed for at least three samples. Antibiotics were only used after transvesical biopsies. In the first year, 149 patients were sampled without any preparation. After three infectious complications in the first year, in the next 154 cases, the biopsy was performed after vaginal disinfection (Octenisept^®^, Schülke^®^, Norderstedt, Germany). In the last 48 cases, a local anaesthetic gel (12 mL Lido C Sterile Catheter Gel, Turkuaz Sağlık Medical Products, Istanbul, Turkey) was also placed into the vagina.

In our studies, we have always considered whether transabdominal or transvaginal sampling would be preferable. In general, a transvaginal examination was preferred due to its better resolution and more precise targeting ability (mass of the adipose tissue, involuntary movement and respiration of the patient are less-limiting factors). Cystic lesions were also sampled. The size of the lesion was not a limiting factor. If the lesion was clearly visible during the ultrasound examination, several attempts were made to obtain a sufficient tissue sample.

### Pathological Procedure

All the sampled core biopsy specimens were immediately fixed in 8% formaldehyde solution and transferred to our Surgical and Molecular Pathological Department on the same day. Based on our experiences for biopsy specimens it is more preferable to use a more diluted formalin (8%) to avoid the possibility of over-fixation and its consequences: decrease of antigenicity or damage of the RNA and DNA content. According to our experiences, this method is reliable; the immunohistochemical reactions and the molecular examinations both function well. All slides were examined by an expert pathologist specialised in gynaecologic oncology (I.V.). Sections from paraffin-embedded tissue blocks were stained with hematoxylin-eosin. Examination of the core biopsy materials had priority in all cases; therefore, processing always began within 24 h. When needed, immunohistochemistry was used to specify the correct histological diagnosis and sometimes to guide therapy. In case of questions or uncertainties, a clinicopathological consultation was organized.

## 3. Results

### 3.1. Results of All 303 Cases

The median age of the 303 patients enrolled was 61 years (21–93 years). The indications for core biopsy sampling were suspicion of primary malignancy in 186 cases (61.4%), suspicion of recurrence in 76 (25.1%), suspicion of metastasis in 13 patients (4.3%), exclusion of malignancy in 21 cases (6.9%) and suspicion of a residual tumour following radiotherapy in 7 cases (2.3%). 136 patients (48.2%) had a previous history of malignancy, among them, 123 (90.4%), 12 (8.9%) and 1 (0.7%) patients had one, two or three different malignant diseases, respectively. A number of 84 (61.8%) of the 136 patients had a history of gynaecological cancer (Table 1). The median number of biopsy cores obtained from each patient was 4 (range: 1–9 cores).

In 299 (98.7%) cases, we obtained adequate specimens; in 1 (0.3%) case a cystic pararectal lesion of 1 cm was not suitable for sampling; in 3 (1.0%) cases the specimen was not suitable for histological diagnosis due to necrosis or insufficient material. The most common site of biopsies was the adnexa (90 cases, 29.7%). Further specimens were obtained from the vaginal stump or wall in 41 (13.5%), the uterus in 35 (11.6%), the peritoneum in 31 (10.2%), the cervix and the parametrium in 28 (9.2%), the parailiac lymph nodes (PIL) in 10 (3.3%), the rectovaginal septum in 9 (3.0%), the bladder in 7 (2.3%), the endometrium in 4 (1.3%) and the retroperitoneum in 1 case (0.3%). In 47 cases (15.5%), sampling was performed on an uncertain pelvic lesion (Table 1). Four patients, where the site of the biopsy was the endometrium itself, were referred from other hospitals to our department after several unsuccessful attempts at conventional endometrial sampling (Table 2). These diagnostic procedures failed due to conglutination of the uterine cervix after multiple conisations in their medical history. TVUS raised the suspicion of endometrial pathology due to thick postmenopausal endometrium. The biopsy confirmed benign histology in 3 patients and in 1 patient, complex hyperplasia of the endometrium with atypia was diagnosed.

Most malignancies had an ovarian origin (103 cases, 34.1%) (Table 2). Other common origins were gastrointestinal (31 cases, 10.3%), cervical (29 cases, 9.6%), endometrial (17 cases, 5.6%), uterine mesenchymal (10 cases, 3.3%) and breast (9 cases, 3.0%). Rarely, haematological (4 cases, 1.3%), vulvar (2 cases, 0.7%), bladder (1 case, 0.3%), skin (1 case, 0.3%) and soft tissue (1 case, 0.3%) was proven to be the primary origin of the malignancy. Nearly one-third of the patients (93 cases, 30.8%) had no malignant lesions. Malignancy was confirmed in two-third of the patients (201/303, 67.3%) based on the core biopsy histology (Table 3). A primary malignancy was diagnosed in 111 of the 201 histologically verified malignant cases (55.2%). Recurrent tumour occurred in 49 (24.4%), metastasis was found in 38 (18.9%) and a residual tumour was detected in 3 cases (1.5%). Interestingly, 23.9% (48/201) of malignant tumours were proven to have a non-gynaecological origin.

The pathologists face difficulties in giving a definitive diagnosis due to the limitations of biopsy samples. In these specimens, only a fraction of the often large and sometimes heterogenic lesions can be seen, and due to the small dimensions, only the minimal required stains can be used. A continuous consultation between pathologist and clinician is needed to decide which predictive markers and molecular methods are used. IHC stains are used neither in cases of parabiopsies nor in most of the unambiguous benign and serous borderline cases (these are about 30% of all cases). Furthermore, in recurrent or metastatic cases (51 cases) only minimal or no stains are needed; in contrast, more IHC stains are needed in most of the malignant and mucinous borderline tumour cases. Using IHC panels depends on the localisation and histomorphology. Table A1 (see Appendix A) summarises the most frequently used IHC stains in different cases without claiming completeness.

#### 3.1.1. Association of Histological Groups with the Biopsy Location

A clear association was found between the site of biopsies and the different histological groups (see Appendix A Table A2). For example, high-grade epithelial ovarian tumour groups occurred most frequently during adnexal (37/83, 44.6%), unspecified pelvic lesions (17/83, 20.5%), peritoneal (19/83, 22.9%) and vaginal stump or wall (7/83, 8.4%) biopsies. Gastrointestinal tumours were most pronounced in adnexa (14/31, 45.2%), unspecified pelvic lesions (6/31, 19.4%), vaginal stump or wall (4/31, 12.9%), rectovaginal septum (3/31, 9.7%), peritoneum (2/31, 6.5%), uterus (1/31, 3.2%) and cervix/parametrium (1/31, 3.2%). Of the 9 breast carcinomas, 7 (77.8%) were found in the ovaries, one (11.1%) in the uterus and one (11.1%) in the parailiac lymph nodes.

#### 3.1.2. Complications

Although all the patients tolerated the procedure well, with a slight modification of the protocol to increase comfort, local anaesthesia was used in the last 42 cases (13.9%). Biopsy was performed under general anaesthesia in 3 patients (1.0%) due to painful postirradiation vaginal stenosis. No complications were observed during the biopsy procedures. There were no cases of bleeding or hematoma in our study. As far as we know, 3 patients (1.0%) developed abscesses. In 2 patients, after the biopsy procedure, cystic lesions became infected. In both patients, though being asymptomatic, surgical exploration confirmed the presence of abscesses in otherwise benign ovarian cystic lesions. In 1 immunosuppressed patient, a large pelvic mesothelioma became superinfected during the procedure, which was resolved by subsequent ultrasound-guided drainage and parenteral antibiotic treatment. One patient (0.3%) died the night after the biopsy due to her advanced disease. She already had a pulmonary embolism and multiplex pulmonary and osseous metastases of suspected ovarian origin. A newly developed severe embolism led to the patient’s death. The core biopsy was taken from the ovaries but the final histology confirmed a fibroma. All 3 infectious complications occurred during the first 149 biopsy (49.2%) procedures when vaginal disinfection was not routinely used. In the following 154 patients (50.8%), routine vaginal disinfection was performed and no more infectious complications occurred.

No prospective data were recorded during the study on the tolerability of the procedure by the patients. Our experience, however, shows that patients’ anxiety plays a major role. The majority of patients reported the sampling was not as painful or uncomfortable as they had imagined. The procedure is quick: the biopsy itself barely lasts about 5 s per sample, and the whole sampling process only takes 1–2 min. In cases where the cul-de-sac peritoneum was not pierced, patients tolerated the sampling without any significant pain. In addition to deep breathing before the puncture, and with the use of a local anaesthetic gel containing lidocaine, further improvement was observed. Consistency of the pierced anatomical structures can also influence the intensity of pain. Based on our experience, a transvesical biopsy is the most painful procedure for patients.

### 3.2. Results of 94 Patients Who Underwent Surgical Exploration

#### 3.2.1. Comparative Analysis of Tru-Cut Biopsy and Surgically Obtained Histological Results (94/303 Cases)

We had 94 patients (31.0%) who underwent surgical exploration following the biopsy procedure. We analysed this group separately in order to determine the true accuracy of the biopsy comparing its result to the final surgical histologic diagnosis. We found histological inaccuracy in 12 patients (12.8%) which will be discussed in detail later. Sensitivity, specificity, positive predictive value (PPV) and negative predictive value (NPV) of the biopsy procedure was 94.8%, 94.1%, 98.6% and 80.0%, respectively (Table 4).

#### 3.2.2. Pathological Evaluation of Inaccurate Histological Results (12/94 Cases)

Our pathologist reviewed 12 cases to analyse the accuracy of the biopsy procedure comparing to the final postoperative histological diagnosis. The main reasons for the discrepancy between the two histological diagnoses were tumour heterogeneity in 6 cases (6.4%), sampling error in 3 cases (3.2%) and differential diagnostic problems in the remaining 3 cases (3.2%).

In general, confirming malignancy from a small amount of specimen obtained by a needle biopsy is always challenging to the pathologist. Those 6 patients whose histological diagnosis was slightly modified after surgical exploration are listed in Table 5. In 4 of these 6 inaccurate cases, the biopsy correctly identified the dignity of the lesions, therefore, the inaccuracies have not influenced the optimal therapy.

In 3 patients, inaccuracy of the results can be attributed to sampling error: in 1 patient, a serous cystadenoma component of the ovarian lesion was represented and a cystadenocarcinoma component was missing from the biopsy core. In another patient, the ovarian core biopsy did not confirm malignancy; however, mesothelioma was detected on the ovarian surface after adnexectomy. In the last case, the ovarian biopsy specimen simply was not informative.

There are cases where the type of tumour cannot be exactly determined from the tissue image; therefore, a molecular pathological examination is required. In 1 case, the lesion corresponded primarily to a fragment of a moderately differentiated Sertoli–Leydig cell tumour based on tissue appearance. However, after the FOXL2 mutation study, the molecular pathological diagnosis confirmed an adult granulosa-cell-type tumour. It should be noted that the differential diagnostic problem that occurred did not affect the patient’s further therapy and no disadvantages arose. In the second case, the possibility of a minimal deviation adenocarcinoma could not be ruled out due to the presence of deeply scattered, non-atypical glands in the cervix and we had no immunohistochemistry to support or exclude the malignancy. However, based on tissue images, it was impressed primarily as a cervix with a retained structure. In the last case, the lesion was considered to be primarily a leiomyogenic tumour with uterine epithelioid morphology in which the possibility of malignancy arose due to epithelioid morphology, cell richness and diffuse p53 positivity (based on which it was considered as a mutant type). Unexpectedly, it turned out there was an interpretive problem in immunohistochemistry causing a differential diagnostic problem. P53 stain reaction was considered positive and mutant in the core biopsy sample; the reaction in the postoperative sample verified a wild-type tumour p53.

Overall, in 8 of the histologically inaccurate cases, the treatment was not affected by this inaccuracy. Deviation from the optimal therapy would have occurred in 4 patients (4.3%) due to an inaccurate histological result. However, based on the clinical picture in these patients, the optimal surgery was performed despite the negative biopsy results.

## 4. Discussion

Adequate histological diagnosis defines the treatment in gynaecologic oncology. Although transvaginal ultrasound is widely used in the diagnosis of pelvic tumours, TVUS-guided core/tru-cut biopsy is not a common procedure. In many centres, computed tomography (CT)-guided needle core biopsy is the gold standard procedure to obtain histology from pelvic malignancies. When its diagnostic performance was studied, the results were similar to those reported in our present study: sensitivity: 84.6%, specificity: 100%, PPV: 100% and NPV: 78.9% [25]. Ultrasound-guided and CT-guided core biopsies are both well-established, minimally invasive methods capable of providing good quality specimens for histological diagnosis. However, the transvaginal route may provide an easier exposure to lesions situated deeply in the pelvic cul-de-sac or the gynaecological organs [5,10,12,14,18,19]. CT-guided procedures are more expensive and their availability is limited in our country; therefore, ultrasound-guided biopsies may accelerate the diagnostic process compared to CT-guided biopsies. In our department, advanced-stage primary gynaecological cancer patients and patients with recurrent pelvic tumours were systematically and quickly referred to expert ultrasound diagnosis and TVUS-guided core biopsy procedure. Systematic integration of this diagnostic activity into our outpatient unit facilitated a one-step ultrasound diagnosis and histologic confirmation of the suspicious pelvic lesions, enabling quick referral to definitive therapy. Likewise, 15.0% of patients were biopsied on the day of their first visit to our institute. Although patients were referred with a suspicion of gynaecological cancer, in 23.9% (48/201), the biopsy confirmed a metastatic non-gynaecologic cancer. In these non-gynaecological tumour cases, unnecessary gynaecological surgery could be avoided and patients could be referred to definitive oncological therapy. Interestingly, in two cases, primary colorectal cancer was diagnosed from a biopsy of the rectovaginal septum. The proportion of non-gynaecological tumours in our study is larger than those reported in the literature [19]. A possible explanation might be that this study was performed in a comprehensive cancer centre with many patients already treated for a malignant condition.

This paper is, to date, the largest reported series of TVUS-guided core biopsy in pelvic tumours and the first one reporting complications following the procedure. Interestingly, the three abscesses after biopsy occurred during the first 149 procedures. When routine, preoperative vaginal disinfection was introduced, no more infectious complications were detected. The utilisation of vaginal routine disinfection is contradictory in the series reported so far [19].

The TVUS-guided core biopsy is an easy-to-use method providing good quality specimens, as adequate histologic specimens could be obtained in 299 patients (98.7%). Comparing the results of the first 4 months (39 cases) to the results of the following period (18 months, 265 cases), using this procedure we could not see a difference in the complication rate, nor in the adequacy of the histologic specimen or the number of false-negative results. This analogy indicates a steep learning curve. Having experience in gynaecological cancer ultrasound examination is essential in TVUS-guided biopsy. The reported biopsies were performed by one investigator (D.L.) and according to our experience, the sampling procedure was confidently mastered within a few weeks, after the first 10 cases. The advantage of our study is that all the biopsies were performed by the same experienced gynaecologist and the data collection was prospective. Another major advantage is that we had the opportunity to compare the histological result of the biopsy to the postoperative histological ones in 94 patients (31.0%). Therefore, we could precisely determine the diagnostic performance of the biopsy procedure, yielding a sensitivity of 94.8% and a specificity of 94.1%, which is considered satisfactory. The calculated NPV was 80%, which might raise awareness: in case of clinical suspicion of malignancy, surgical exploration is suggested despite a negative biopsy result, as false negativity cannot be ruled out. This performance is similar to the literature previously reported; however, to the best of our knowledge, this study is the largest comparison between the transvaginal biopsy and surgical histological results [1,18,19]. Due to the heterogeneity of many gynaecological tumours, we believe a core biopsy might be advantageous over a fine needle aspiration biopsy because it provides more detailed information about tissue structure. Diagnostic and prognostic immunohistochemical and molecular examinations can be performed from the sample; moreover, they can guide therapeutic decisions [21,26,27,28]. Still, tumour heterogeneity resulted in inaccurate histological diagnosis in 6 of the 94 analysed patients. One limitation of our study is the fact that our department is part of a comprehensive cancer centre; therefore, our results cannot be simply translated to other general gynaecological departments. Another limitation is the lack of central pathological review; however, all the samples were evaluated by the same experienced gynaeco-pathologist.

## 5. Conclusions

According to our experience, TVUS-guided NCB is a safe and effective histological sampling procedure, providing adequate tissue for pathological evaluation in 99% of cases. It can reliably guide therapy as its performance is satisfactory compared to surgically obtained histology. As infectious complications might rarely occur, routine preoperative vaginal disinfection is suggested. In case of the suspicion of malignancy despite negative biopsy histology, further investigation is proposed due to the 80% NPV. TVUS-guided core biopsy is an effective diagnostic method providing possible benefit to patients with a suspicion of gynaecological malignancy.

## Figures and Tables

**Table 1:** Characteristics of the patients included in the study.

Parameter	Value (*n* = 303)
Median age (yr) (range)	61 (21–93)
Median biopsy core number (range)	4 (1–9)
Indication of biopsy sampling	-
Suspicion of a primary cancerous process	186 (61.4%)
Suspicion of recurrence	76 (25.1%)
Exclusion of malignancy	21 (6.9%)
Suspicion of metastasis	13 (4.3%)
Suspicion of a residual tumour	7 (2.3%)
Previous history of malignancy	-
One previous malignant tumour	123 (40.6%)
Two previous malignant tumours	12 (4.0%)
More than two previous malignant tumours	1 (0.3%)
No known previous malignant tumour	167 (55.1%)
Previous history of gynaecological cancer	-
Yes	84 (27.7%)
No	219 (72.3%)
Site of biopsy	-
Adnexa	90 (29.7%)
Vaginal stump or wall	41 (13.5%)
Uterus	35 (11.6%)
Peritoneum	31 (10.2%)
Cervix/parametrium	28 (9.2%)
Parailiac lymph nodes (PIL)	10 (3.3%)
Rectovaginal septum	9 (3.0%)
Bladder	7 (2.3%)
Endometrium	4 (1.3%)
Retroperitoneum	1 (0.3%)
Uncertain pelvic lesion	47 (15.5%)
Preliminary imaging	-
CT	155 (51.2%)
US	67 (22.1%)
MRI	58 (19.1%)
PET-CT	23 (7.6%)

**Table 2:** Summarized results of the biopsies.

Parameter	Value (*n* = 303)
Adequate histology obtained (patients)	299 (98.7%)
Adequate histology not obtained (patients)	4 (1.3%)
Median elapsed days between sampling and histological diagnosis (range)	7 (3–26)
Median number of immunohistochemical (IHC) reactions used for all histology samples (range)	2 (0–13)
Median number of IHC reactions by histological groups (range)	-
High-grade epithelial ovarian carcinoma	2 (0–12)
Low-grade epithelial ovarian carcinoma	4.5 (0–9)
Non-epithelial ovarian carcinoma	6 (2–9)
Low-grade endometrial carcinoma	5 (0–8)
High-grade endometrial carcinoma and carcinosarcoma	5 (3–12)
Malignant uterine mesenchymal tumour	5 (4–12)
Cervical squamous cell carcinoma	2 (0–11)
Cervical adenocarcinoma	7 (4–10)
Gastrointestinal tumour	4 (2–12)
Breast carcinoma	8 (3–13)
Vulva	0 (0–0)
Other malignant	6.5 (2–10)
Unknown	6 (4–10)
All benign	0 (0–13)
Primary origin of the malignant lesion	-
Ovarian	103 (34.1%)
Gastrointestinal	31 (10.3%)
Cervical	29 (9.6%)
Endometrial	17 (5.6%)
Uterine mesenchymal	10 (3.3%)
Breast	9 (3.0%)
Haematological	4 (1.3%
Vulvar	2 (0.7%)
Bladder	1 (0.3%)
Skin	1 (0.3%)
Soft tissue	1 (0.3%)
Unknown (neuroendocrine)	1 (0.3%)
Non-malignant	93 (30.8%)
Types of malignancy	-
Primary tumour	111 (55.2%)
Recurrence	49 (24.4%)
Metastasis	38 (18.9%)
Residual tumour	3 (1.5%)
Further subsequent treatment	-
Chemotherapy	107 (35.3%)
Surgery	72 (23.8%)
Observation	30 (9.9%)
None	15 (5.0%)
Radiotherapy	12 (4.0%)
Best supportive care	5 (1.7%)
No information	62 (20.5%)

**Table 3:** Characteristics of the histological groups.

Histological Groups/Types of Malignancy (*n* = 201)	Primary Tumour	Metastasis	Recurrence	Residual Tumour
High-grade epithelial ovarian carcinoma	77	0	6	0
Low-grade epithelial ovarian carcinoma	6	1	5	0
Non-epithelial ovarian carcinoma	3	0	3	0
Low-grade endometrial carcinoma	4	0	5	0
High-grade endometrial carcinoma and carcinosarcoma	8	0	2	0
Malignant uterine mesenchymal tumour	2	0	2	0
Cervical squamous cell carcinoma	6	1	13	3
Cervical adenocarcinoma	1	1	2	0
Gastrointestinal tumour	2	22	7	0
Breast carcinoma	0	6	3	0
Vulva	0	2	0	0
Other	2	5	1	0

**Table 4:** Comparative analysis of tru-cut biopsy and surgically obtained histological results.

	Surgery: Malignant	Surgery: Benign
Biopsy: malignant	73 (True malignant)	1 (False positive)
Biopsy: benign	4 (False negative)	16 (True benign)

**Table 5:** Histologic results of the heterogeneity.

Histology of Biopsy	Histology of Surgery
Undifferentiated endometrial carcinoma	Serous endometrial carcinoma
Thecoma	Granulosa cell tumour
Serous borderline tumour	Low-grade serous tumour
Borderline clear cell tumour	Invasive clear cell carcinoma
Endometrial complex hyperplasia without atypia	Well-differentiated endometrial carcinoma
Suspicion of malignant soft tissue tumour	Immature teratoma

## Data Availability

The dataset used in this study is not available online. This is available on request from the corresponding author.

## Appendix A

**Table A1:** Immunohistochemical stains used in gynaecological tumours.

Localization	Types of Tumours	Subtypes	Stains (Considering the Clinical Data and Histomorphology)
Adnexa/peritoneum	Undifferentiated tumour	-	Basic panel: AE1/AE3, CK7, CK8/18, SALL4, Vimentin, LCA, PAX8, SOX10 (further IH stains depend on the results)
-	Epithelial tumour	-	-
-	-	Suspicion of metastatic epithelial tumour	CK7, CK20, CDX2 (CDH17, SATB2), PAX8, TTF1, GATA3
-	-	Serous	PAX8, WT1, p53
-	-	Mucinous	CK7, CK20, CDX2, (CDH17, SATB2), PAX8, ER
-	-	Endometrioid	(Sometimes exclusionary diagnosis): CK7, CK20, CDX2, PAX8, WT1, p53, ER, (p63, CD10, SATB2: morula formation) (clinical data to rule out metastasis of endometrial origin)
-	-	Clear cell	PAX8, ER, WT1, p53, HNF1ß (clinical data to rule out metastasis of renal or endometrial origin)
-	-	Brenner tumour	GATA3, CK7, CK20, p63 (clinical data to rule out metastasis of urothelial origin)
-	-	MMMT	p53 (furthermore, it depends on its components if it is necessary) (clinical data to rule out metastasis of endometrial origin)
-	Stromal tumour	-	inhibin, calretinin, CK, WT1, FOXL2 or Dicer 1 mutation analysis
-	Germ cell tumour	-	SALL4, OCT3/4, PLAP (D240, ckit), CD30, AFP, (GATA3, HNF1ß), ß-HCG, hPL, CK7, GATA3, in case of teratoma, it depends on the components
Cervix	Squamous cell carcinoma	-	(p63, p16)
Cervix/endometrium	adenocarcinoma	HPV-associated	p16, p53, ER, PR, Vimentin, (HPV-analysing)
-	-	non-HPV-associated	p16, p53, ER, PR, MMR proteins, (other type-specific markers, such as MUC6, GATA3, calretinin, CD10, TTF1, HNF1ß, CD56, ChrA, Synaptophysin) (clinical data to confirm the exact origin)
Endometrium	Adenocarcinoma	-	p16, p53, ER, PR, CK, PAX8 (avoiding to confuse serous or dedifferentiated carcinoma with grade I–II endometrioid carcinoma)
mesenchymal tumours	Stroma and leiomyoma tumours	-	CD10, Cyclin D1, SMA, desmin, h-caldesmon, ER, PR, p16, p53, Ki-67

Abbreviations: AFP: alfa-fetoprotein, CD: cluster of differentiation, CDH17: cadherin-17, CDX2: caudal-type homeobox 2, CK: cytokeratin, GATA3: GATA (guanine, adenine, thymine, adenine)-binding protein 3, ER: estrogen receptor, FOXL2: forkhead box protein L2, ß-HCG: human choriogonadotropin, HNF1ß: hepatocyte nuclear factor, hPL: human placental lactogen, HPV: human papillomavirus, IH: immunohistochemical stains, LCA: leukocyte common antigen, MMMT: malignant mixed üllerian tumours, MMR: mismatch repair, MUC6: mucin 6, OCT3/4: organic cation transporter 3/4, PAX8: paired box gene 8, PLAP: placental alkaline phosphatase, PR: progesterone receptor, SALL4: Sal-like protein 4, STB2: special AT (adenine, thymine)-rich sequence-binding protein 2, SOX10: SRY (sex-determining region Y)-related HMG ((high mobility group)-box 10, TTF1: Thyroid transcription factor 1, WT1: Wilms’ tumour protein 1.

**Table A2:** Association of histological groups with the site of biopsy (PIL: Parailiac lymph node).

Histological Groups/Sampling Site (*n*)	Adnexa	Uncertain	Rectovaginal Spatium	Peritoneum	Endometrium	Uterus	PIL	Cervix/ Parametrium	Bladder	Vaginal Stump or Wall	Retroperitoneum
High-grade epithelial ovarian carcinoma	37	17	2	19	0	0	1	0	0	7	0
Low-grade epithelial ovarian carcinoma	4	2	0	1	0	0	0	0	1	4	0
Non-epithelial ovarian carcinoma	3	1	0	1	0	0	0	0	0	1	0
Low-grade endometrial carcinoma	1	2	0	0	0	2	0	0	0	4	0
High-grade endometrial carcinoma (carcinosarcoma too)	1	1	0	0	0	5	0	1	0	2	0
Malignant mesenchymal corpus tumour	0	1	0	0	0	1	0	0	0	2	0
Cervical squamous cell tumour	0	1	1	1	0	2	1	12	0	5	0
Cervical adenocarcinoma	0	0	0	0	0	0	2	0	0	2	0
Gastrointestinal tumour	14	6	3	2	0	1	0	1	0	4	0
Breast tumour	7	0	0	0	0	1	1	0	0	0	0
Other	3	1	0	1	0	0	2	0	1	0	0
Benign	16	11	1	1	1	16	1	1	1	2	1
Sine morbo	3	2	0	2	3	7	1	12	4	8	0
Vulva	0	0	1	0	0	0	1	0	0	0	0
Uncertain	1	2	0	3	0	0	0	1	0	0	0
